# Acknowledgment of Reviewers, 2020

**DOI:** 10.1200/GO.20.00349

**Published:** 2020-08-12

Peer review is at the core of scientific progress. It is through the careful critique of peer reviewers that papers are scrutinized, evaluated, and improved. A paper that has been peer reviewed bears the mark of having been inspected and found to be valid and worthy of publication. It is these peer-reviewed papers that ultimately advance our existing understanding of science.

As such, scientific outlets in general, and *JCO Global Oncology* in particular, owe an immense debt of gratitude to peer reviewers. *JCO GO* strives to provide the highest quality peer review, so in recognition of Peer Review Week 2020 and its theme of Trust in Peer Review, we are pleased to recognize your efforts and thank you for your expertise and time by presenting this list of those who have contributed to our success.

For your hard work in 2019-2020, our heartfelt thank you!

An asterisk indicates reviewers who have reviewed two or more manuscripts in 2019-2020. Two asterisks indicate reviewers who completed the ASCO Reviewer Mentoring program. Three asterisks indicate reviewers who participated in the ASCO Editorial Fellowship program.

Innocent Abayo

Raafat Abdel-Malek*

Hikmat Abdel-Razeq

Judith Abrams*

Maria Abriata

Nour Abuhadra**

Dafalla Abuidris

Aleesha Adatia*

Babatunde Adedokun

Bolanle Adegboyega

Eric Adjei Boakye**

Aimaz Afrough**

Philippe Aftimos

David Agom

Pedro Aguiar Junior

Fahad Ahmed

Joanne Aitken

Oladimeji Akinboro

Murat Akova

Sercan Aksoy

Shahinoor Aktev

Samer Al Hadidi**

Thierry Alcindor

Mazin Aljadiry

Gustavo Almeida

Amin Altijani

Christian Alvarez Privado

Nada Alwan

Richard Ambinder

Kate Anderson

Benjamin Anderson

Rifat Ara

Sanchia Aranda*

Avan Armaghani

Simeon Aruah

Banu Arun

Daniel Zemenfes Ashebir

Arwa Ashoor**

Patricia Ashton-Prolla

Nissa Askins

Bassel Atassi

Aaron Atkinson

Kirsten Austad

Shivanshu Awasthi

Burça Aydin

Govind Babu

Ludwing Bacon Fonseca

Ahmed Badawy

Bhausaheb Bagal

Marieke Bak

Pedro Barata

Sanaa Bardaweel

Íbrahim Barişta

Jill Barnholtz-Sloan

Atul Batra

Ann Marie Beddoe*

Asim Belgaumi

Sara Benitez Majano

Jessica Bensenhaver

Safedin Beqaj

Anouk Berger

Alejandro Berlin*

Scott Berry

Pascal Bessong

Nickhill Bhakta*

Rohini Bhatia

Ami Bhatt

Gouri Shankar Bhattacharya*

Barri Blauvelt*

Prunella Blinman

Amy Body

Renata Bonadio

Christopher Booth

Maarten Bosland

Fouad Boulos

Maria Bourlon*, ***

Marouen Braham

Ari Brooks*

Ewan Brown

Henriette Burger*

Sahai Burrowes

Sita Bushan**

Anna Cabanes*

Conceicao Campos**

Saul Campos-Gomez

Eduardo Cazap*

Robert Chamberlain

Zhi Cheng

Alberto Chiappori

Supriya Chopra*

Melvin Chua

YuJo Chua

Linus Chuang*

Harrison Chuwa

Marcia Ciccone

Mishka Cira

Christina Clarke

Graham Colditz

C. Norman Coleman*

Brian Collopy

Michelle Connolly

Olivia Cook*

Ainhoa Costas-Chavarri

Joanna Coutts

Mel Valerie Cruz-Ordinario

Alberto Jacobo Cunquero Tomás**

Giuseppe Curigliano

Sanju Cyriac

Christopher Dandoy

H.S. Darling*

Amy Davies

Thomas Davis

Daphne Day*

Gilberto de Castro Junior

Alba de Jesús Kihn Alarcón***

Paul de Souza

Rejane de souza Reis

Kenny Jun Demegillo

Lynette Denny

Nebiyu Dereje

Aakash Desai

Amrita Desai

Begoña Díaz de la Noval**

Michelle Dickson

Helen Dimaras

Julia Downing

Arielle Eagan

Nagi El Saghir

Elena Elimova

Yannick Eller

Ahmed Elzawawy*

Temidayo Fadelu

Yufei Fan

Muhammad Mohsin Fareed**

Fadi Farhat

Tamer Fouad

Jane Fox

Lewis Foxhall

Marie Belle Francia

Lindsay Frazier

Shridar Ganesan

Trivadi Ganesan

Marina Garassino

Arun Garg*

Benjamin Garmezy**

Gemma Gatta

Fabio Girardi

Meredith Giuliani*

Daniel Goldstein

Annekathryn Goodman

Dan Green Renner

Rajesh Grover

Sumit Gupta

Jason Gurney*

Bishal Gyawali*

Neville Hacker

Ezra Hahn

Khalid Halahleh

Evan Hall

Daniel Haller

Nazik Hammad

Kathy Han

Rachel Hanisch

Timothy Hanna

Prashanth Hari Dass

Stephen Hawes

Alexander Heriot

Peter Hesseling

Ekkehard Hewer

Jini Hyun**

André Ilbawi

Mary Isaacson

Muhammad Islam

Sharif Ísmail

Christopher Jackson*

Abdul-Rahman Jazieh

Ahmedin Jemal

Deepa Joseph*

Dilyara Kaidarova

Aruna Kamimeni

Violet Kayamba

Jamal Khader

Uqba Khan**

Nukhet Kirag

Eric Krakauer

Robert Kridel

Nicole M. Kuderer

Jagadish Kudkuli

Vishal Kukreti

Shaji Kumar

Rajat Kumar

John Kuruvilla

Bahar Laderian**

Iissam Lalya

Arjun Law

Rita Lawlor

Angeline Letendre

Ditte S. Lind

Jocelyn Lippey*

Jeffrey H. Lipton*

T. Andrew Lister*

Karolina Lisy

Patrick Loehrer

Dorothy Lombe*, **

Sandra Luna-Fineman

Vijay Mahobia

Adrianne Mallen**

Max Mano

Alejandro Marinos Velarde**

Pritchard Mark

Deborah Maskens

Maura Massimino

Charbel Matar*, ***

Aju Mathew

Gustavo Matos**

Amelia McCartney

Itrat Mehdi

Parth Mehta

Les Mery

Monika Metzger

Vivienne Milch*

Brittany Miller

Dan Milner

Lori Minasian

Mark Minden

Michelle Mollica

Mariana Monteiro

Lesley Moody

Fabio Moraes*

Dion Morton

Deborah Mukherji

Pablo Munoz Schuffenegger

Gad Murenzi

Tracy Murphy

Shilpa Murthy

Miriam Mutebi

Alex Mutombo Baleka

Nataraj Naidu

Steven Narod

Gaurav Narula

Kingsley Ndoh

Sweet Ping Ng**

Mike Nguyen

Tu Nguyen-Dumont

Wilfred Ngwa*

Claire Nightingale*

Albert Nyamhunga

Folakemi Odedina*

Samson Okello

Olufunmilayo Olopade

Maria Orozco

Lydia Pace

Matthew Painschab

Robert Parker

Nir Peled

Edith Perez

Philip Philip

Leeya Pinder

Chelsea Pinnix*

Hans Prenen

Ajay Raghunath

Kanti Rai

Jordi Remon

Raul Ribeiro

Eli Ristevski

Mark Robson

Danielle Rodin

Lindsey Roeker

Paul Rogers

Anya Romanoff

Davide Rossi

Sevket Ruacan

Mark Rutherford

Danielle Benedict Sacdalan**

Adrian Sacher

Muhammad Saghir

Arzu Saglam

Rakiya Saidu

Suraj Samtani**

Marceli Santos

Kartik Sehgal

Meltem Sengelen*

Jaime Shalkow

Ramila Shilpakar*, ***

Rajesh Shinde**

David Shultz

Hannah Simonds*

George Sledge

Jeremy Slone

Robert Smith

Angela Solano

Montserrat Soler

Enrique Soto-Perez-de-Celis*

Priyanka Srivastava

Susannah Stanway

Daniela Stefan

Simona Stolnicu

Simone Strasser

Daniel Surridge

Gevorg Tamamyan

Han Chong Toh

Edward Trimble*

Derek Tsang

Vivien Tsu

Siddharth Turkar**

Jayant Vaidya

Jacob Van Dyk*

Verna Vanderpuye*

Vinicius Vazquez

Christopher Venner

E. Vidhubala

Katherine Virgo

Auro Viswabandya

Daniel Vorobiof

Horia Vulpe*

Sara Wahlroos

John Waldron

Kate Webber

Jeffrey Weitzel

Brooke Wilson

Paul Wissel

Rebecca Wong*

Suayib Yalcin

Jennifer Yeh

Desmond Yip

Roberto Zanetti*

Nik Zeps

Eduardo Zubizarreta*

Jo Anne Zujewski

